# BMP signaling balances proliferation and differentiation of muscle satellite cell descendants

**DOI:** 10.1186/1471-2121-12-26

**Published:** 2011-06-06

**Authors:** Melanie Friedrichs, Florian Wirsdöerfer, Stefanie B Flohé, Sabine Schneider, Manuela Wuelling, Andrea Vortkamp

**Affiliations:** 1 Center for Medical Biotechnology, Faculty of Biology, Department of Developmental Biology, University of Duisburg-Essen, D-45117 Essen, Germany; 2 Department of Paediatrics, University Hospital of Essen, D-45147 Essen, Germany; 3 Surgical Research, Department of Trauma Surgery, University Hospital Essen, D-45147 Essen, Germany

## Abstract

**Background:**

The capacity of muscle to grow or to regenerate after damage is provided by adult stem cells, so called satellite cells, which are located under the basement lamina of each myofiber. Upon activation satellite cells enter the cell cycle, proliferate and differentiate into myoblasts, which fuse to injured myofibers or form new fibers. These processes are tightly controlled by many growth factors.

**Results:**

Here we investigate the role of bone morphogenetic proteins (BMPs) during satellite cell differentiation. Unlike the myogenic C2C12 cell line, primary satellite cells do not differentiate into osteoblasts upon BMP signaling. Instead BMP signaling inhibits myogenic differentiation of primary satellite cells *ex vivo*. In contrast, inhibition of BMP signaling results in cell cycle exit, followed by enhanced myoblast differentiation and myotube formation. Using an *in vivo *trauma model we demonstrate that satellite cells respond to BMP signals during the regeneration process. Interestingly, we found the BMP inhibitor *Chordin *upregulated in primary satellite cell cultures and in regenerating muscles. In both systems *Chordin *expression follows that of Myogenin, a marker for cells committed to differentiation.

**Conclusion:**

Our data indicate that BMP signaling plays a critical role in balancing proliferation and differentiation of activated satellite cells and their descendants. Initially, BMP signals maintain satellite cells descendants in a proliferating state thereby expanding cell numbers. After cells are committed to differentiate they upregulate the expression of the BMP inhibitor *Chordin *thereby supporting terminal differentiation and myotube formation in a negative feedback mechanism.

## Background

Adult muscle tissue is characterized by its capacity to grow after exercise and to regenerate after injury. Both processes require stem cells - so called satellite cells, which are located between the myofiber membrane and the surrounding basement lamina [1]. Activation of quiescent satellite cells by external stimuli, like exercise or injury, leads to their re-entry into the cell cycle to provide myoblasts to the regenerating muscle. While most of the satellite cell descendants are committed to differentiate, either by fusing to existing fibers or by forming new myofibers, some of these cells do not enter the differentiation program, but exit the cell cycle and return to a quiescent state [2]. The activated satellite cells will thus not only provide myonuclei to the regenerating muscle but will also replenish the stem cell reservoir [3-5].

During satellite cell differentiation distinct phases can be distinguished, which are characterized by the expression of different transcription factors. The paired-box transcription factor Pax7, a marker widely used to identify satellite cells, is expressed in quiescent satellite cells [6]. Activated satellite cells maintain Pax7 expression and, in parallel, upregulate MyoD, a member of the family of myogenic regulatory factors (MRFs). MyoD expression is maintained during proliferation and early differentiation [5,7-10]. Commitment to differentiation is characterized by the onset of Myogenin expression, another member of the group of MRFs [5,7-10]. Upon terminal differentiation postmitotic satellite cell descendants fuse into myotubes and start to express muscle structure proteins like myosin. Based on the expression of these transcription factors adult regenerative myogenesis largely recapitulates the embryonic and fetal program of muscle differentiation.

During the last years various growth factors and signaling pathways have been identified that control myoblast differentiation during embryogenesis. The process of self-renewal, for example, has been linked to canonical Wnt signaling as well as to the non-canonical planar cell polarity pathway [11,12].

Hepatocyte growth factor (Hgf) is one of the key regulators during activation of quiescent satellite cells. Different studies demonstrate that Hgf, released from the muscle extracellular matrix immediately after injury, signals to the Hgf receptor c-met, which is expressed in quiescent satellite cells [13-15]. The expansion of the satellite cell pool is positively regulated by Fibroblast growth factors (FGFs), which activate proliferation synergistically to Hgf [16]. Furthermore, the expression of Fgf receptor 4 (Fgfr4) has been reported to be upregulated during satellite cell activation [17]. Besides a potential role during satellite cell activation, FGFs control proliferation of satellite cells *in vitro *and *in vivo *[10,18].

Another important group of signaling factors regulating satellite cell differentiation are members of the Transforming growth factor β (TGFβ) family. Tgfβ1, the best-characterized family member during adult myogenesis, has been shown to inhibit satellite cell proliferation and differentiation *in vitro *and to negatively regulate muscle growth and regeneration *in vivo*[19]. TGFβ signaling requires binding of the ligand to type I and type II receptors, leading to the formation of a hetero-tetrameric receptor complex. Phosphorylation of type I by type II receptors leads to the subsequent phosphorylation of receptor regulated SMAD proteins, which form heterodimers with the common coactivator Smad 4. These complexes translocate to the nucleus, where they function as transcription factors to regulate TGFβ target gene expression [20].

Bone morphogenetic proteins (BMPs) form a subgroup of the TGFβ family of growth factors. BMPs regulate target gene expression via phosphorylation of Smad 1, 5 and 8. The activity of BMPs is negatively regulated by secreted BMP inhibitors, like Noggin and Chordin, which inhibit binding of the growth factors to their receptors [21]. BMPs have originally been identified by their capacity to induce bone formation when injected into adult rat muscles [22]. The osteogenic effect of BMPs was intensively studied in C2C12 cells, a cell line derived from regenerating muscle. C2C12 cells will form multinucleated myotubes upon serum starvation, but will differentiate into osteoblasts upon stimulation with BMPs [23-25]. Due to this observation BMP signaling has not been intensively studied in the context of adult myogenesis. In contrast to these cell culture experiments several studies have identified BMPs as regulators of chick embryonic myogenesis in somites and limbs [26-28]. Based on these findings we have re-analyzed the role of BMP signals during satellite cell differentiation using C2C12 cells and primary adult mouse satellite cells. We found that BMPs keep satellite cell descendants in a proliferating state, while cell cycle exit and myotube formation is induced by inhibition of BMP signaling. We also demonstrated that the BMP pathway is activated during *in vivo *muscle regeneration.

## Results

### Inhibition of BMP signaling triggers myogenic differentiation in C2C12 cells

**Figure 1:**
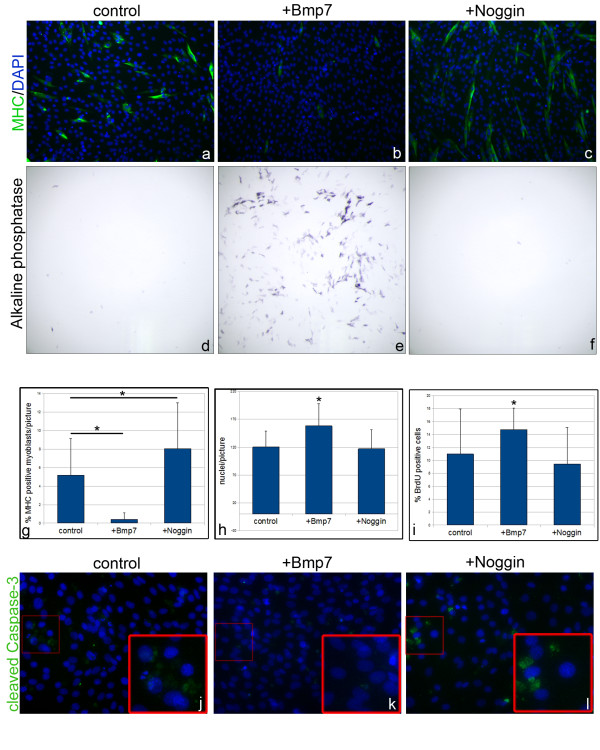
**Bmp7 and Noggin regulate terminal differentiation of C2C12 cells in opposite ways**. C2C12 cells were treated in control medium (A,D,J) or supplemented with Bmp7 (B,E,K) or Noggin (C,F,L) and analyzed for terminal myogenic differentiation (MHC expression) (A-C), osteoblast differentiation (alkaline phosphatase activity) (D-F), BrdU incorporation (I) and apoptosis (J-L). (A-C) terminally differentiated, MHC expressing myoblasts/myotubes can be detected in control cultures (A). Bmp7 treatment inhibits myogenic differentiation (B), whereas Noggin treatment leads to enhanced myotube formation (C). DAPI was used to counterstain nuclei (100× magnification). (D-F) C2C12 cells express alkaline phosphatase upon Bmp7 treatment (E) but not in untreated (D) or Noggin-treated cultures (F) (50× magnification). (G) Statistical analysis using the generalized Wilcoxon test demonstrates significantly reduced numbers of MHC-positive cells after Bmp7 treatment and increased numbers after Noggin treatment (n = 6, 2 individual experiments in triplicates; *p < 0.005). Cell number (H) and BrdU incorporation (I) are upregulated after Bmp7 treatment (n = 6, 2 individual experiments in triplicates *p < 0.005; Wilcoxon test). (J-L) the apoptotic marker cleaved Caspase3 is absent after Bmp7 treatment (K) while apoptosis can be detected in control (J) and Noggin-treated cultures (L) (200× magnification).

To gain first insight into a potential role of BMP signaling during satellite cell differentiation we examined myogenic differentiation of C2C12 cells after treatment with Bmp7 and Noggin (Figure 1; n = 6). As C2C12 cells have been described to transdifferentiate into osteoblasts if treated with BMPs we first monitored the expression of alkaline phosphatase, which is expressed in osteoblasts but not in cells of the myogenic lineage (Figure 1D-F). As expected, a subset of cells treated with Bmp7 for 3 days showed enzymatic activity of alkaline phosphatase (Figure 1E) [25]. In contrast, in untreated or Noggin-treated cultures no differentiation of alkaline phosphatase expressing osteoblasts could be detected (Figure 1D, F). We next analyzed the extent of myogenic differentiation by immunostaining with the muscle myosin heavy chain (MHC) antibody A4.1025, which recognizes all isoforms of skeletal muscle MHCs in terminally differentiated myoblasts (Figure 1A-C,G). In Bmp7-treated cultures only single myoblasts expressed MHC and myogenic differentiation was significantly decreased (0.5%) compared to untreated cultures (5%; p < 0.005). Interestingly, in comparison to control cultures, Noggin-treated cultures showed significantly increased numbers of MHC expressing cells and enhanced myotube formation (8%; p < 0.005).

Besides the inhibitory effect on myogenic differentiation, Bmp7 treatment led to a significantly increased cell number in C2C12 cultures compared to non-treated and Noggin-treated cultures (130%; p < 0.005; Figure 1H). To test if Bmp7 regulates proliferation of C2C12 cells, thereby preventing cell cycle exit and myogenic differentiation, we measured 5-bromo-2'-deoxyuridine (BrdU) incorporation during S-phase after 3 days in culture. (Figure 1I). After treatment with Bmp7, the proportion of cells in S-phase was increased to 15% compared to 11% in untreated and 9.5% in Noggin-treated cultures (Figure 1I) (p < 0.05) [29]. In addition to the reduced number of cells in S-phase, numerous cells detached from the culture well within control and Noggin-treated cultures. To investigate if apoptosis is induced during differentiation of C2C12 cells due to growth factor starvation, we analyzed the presence of cleaved Caspase-3, which triggers the apoptotic process [30,31]. While cleaved Caspase-3-positive, apoptotic cells were detected within the non-treated and Noggin-treated cultures, hardly any apoptotic cells could be detected after Bmp7 treatment (Figure 1J-L) [32]. In summary, the increased cell density in combination with the increased number of cells in S-phase and the reduced number of MHC- and caspase-3-positive cells indicate that Bmp7 signaling protects C2C12 cells from differentiation and apoptosis keeping them in a proliferating state. In contrast inhibition of BMP signaling triggers myogenic differentiation, thereby reducing the number of cells in S-phase.

### Activated satellite cells respond to BMP signals

**Figure 2:**
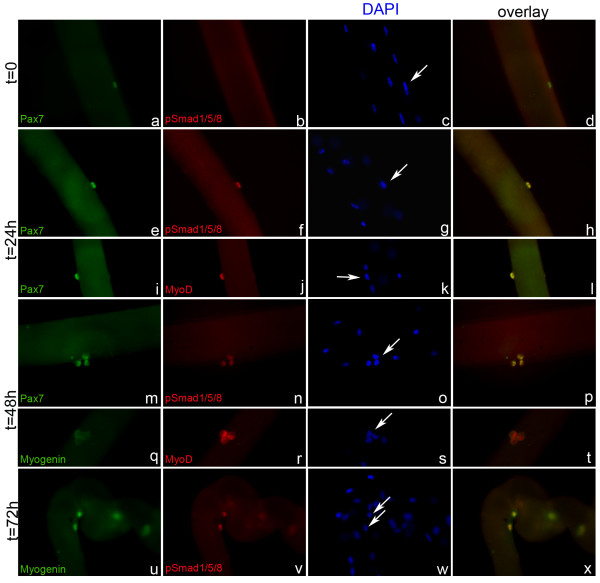
**Activated satellite cells respond to BMP signals**. Satellite cells attached to myofibers were analyzed after 0 (A-D), 24 (E-L), 48 (M-T) and 72 hours (U-X) in culture by co-immunostaining for Pax7 (A,E,I,M), myogenin (Q,U), MyoD (J,R) sein and p-Smad 1/5/8 (B,F,N,V) as indicated. (A-D) p-Smads are absent in quiescent satellite cells. (E-L) Satellite cells co-express Pax7, MyoD and p-Smads after 24 hours. (M-T) after 48 hours Pax7 and MyoD are co-expressed with p-Smad, while Myogenin is absent. (U-X) Myogenin colocalizes with p-Smad after 72 hours. Nuclei were counterstained with DAPI. Magnification is 200×.

As myogenic differentiation is regulated by BMP signaling in C2C12 cells we next asked if satellite cells react to BMP signals *ex vivo *in a similar way. To address this question, we cultured primary satellite cells for 0, 24, 48 and 72 hours on floating myofibers and analyzed the expression of the well-characterized, stage specific transcription factors Pax7, MyoD and Myogenin in relation to BMP induced phosphorylation of Smad 1/5/8 (p-Smad) by double immunolabeling (Figure 2) [5,33,34]. As previously described, quiescent satellite cells on freshly isolated myofibers express Pax7 only (Figure 2A-D). After 24 hours in culture, a time point at which most satellite cells have not yet divided, Pax7 co-localized with MyoD, which marks activated, proliferating and early differentiating satellite cells [34] (Figure 2I-L). At 48 hours, many satellite cells had completed their first cell divisions and expressed Pax7 and MyoD, but not the differentiation marker Myogenin, which marks myoblasts commited to differentiate (Figure 2M-P, Q-T). First nuclear Myogenin could be detected after 72 hours (Figure 2U-X) [5]. We did not detect any p-Smad-positive satellite cells on freshly isolated myofibers (n > 50; Figure 2A-D). Furthermore, no p-Smad staining could be detected in the myonuclei of the fiber (Figure 2A-H, M-P, U-X). At 24 and 48 hours, when satellite cell descendants were still proliferating and had not yet started to differentiate (Myogenin-negative; Figure 2Q-T), all Pax7-positive, MyoD-positive satellite cells were positive for p-Smad (n > 50; Figure 2E-H, M-P). At 72 hours when cells became committed to differentiate, p-Smad and Myogenin colocalize in a subset of cells (Figure 2U-X). Together these data indicate that quiescent satellite cells do not react to BMP signals, but respond to BMP signals upon activation. Furthermore, BMP responsiveness is maintained during early differentiation stages, but is absent in terminally differentiated myonuclei of the fiber.

### BMP signals maintain expression of Pax7 in satellite cells

**Figure 3:**
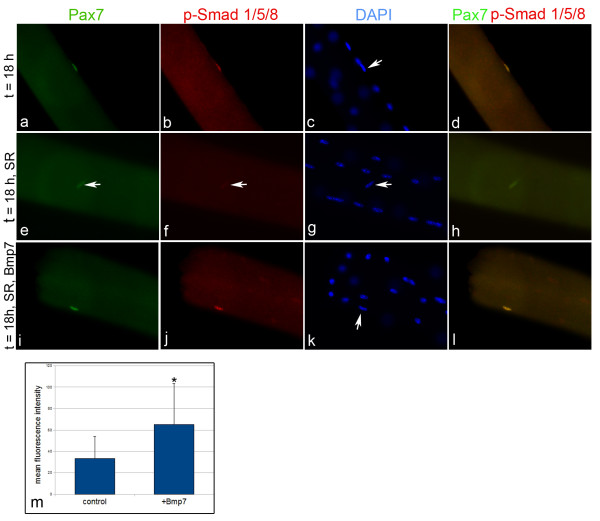
**Bmp7 maintains Pax7 expression in satellite cells**. Satellite cells were cultured on myofibers for 18 hours under standard (A-D) or serum free (SR) (E-L) conditions. (A-D) p-Smad colocalize with Pax7 in standard medium. (I-L) In serum free cultures Bmp7 treatment induces p-Smad phosphorylation in Pax7-positive cells. In untreated cultures p-Smad cannot be detected (E-H). Absence of p-Smad causes a severe downregulation of Pax7. Nuclei were counterstained with DAPI. Magnification is 400×. (M) Analysis of the mean fluorescent intensity of the Pax7 signal in satellite cells revealed a downregulation to 50% in the absence of Bmp7 treatment (n = 30 satellite cells per treatment from 3 individual experiments, * p = 0.005, Wilcoxon test). Magnification is 400×. SR = serum replacement.

Satellite cells grown *in vitro *are exposed to many growth factors present in the culture medium. To test if freshly isolated satellite cells respond to BMP signals directly or if the competence to respond requires other growth factors, we cultivated satellite cells on myofibers under serum-free conditions and treated with Bmp7 (Figure 3; n = 3). After 18 hours in culture p-Smad-positive cells were present on each myofiber treated with Bmp7 (Figure 3I-L). Similar to satellite cells cultured in growth factor rich medium, p-Smad colocalized with Pax7 upon Bmp7 treatment under serum-free conditions (Figure 3A-D, I-L). In contrast, on fibers, which did not receive Bmp7 under serum free conditions, we could hardly detect nuclear p-Smad in satellite cells (Figure 3E-H). Surprisingly, in these untreated cells the expression of Pax7, the marker for quiescent satellite cells, was strongly reduced, (Figure 3E-H). Quantification of the fluorescence intensity of the Pax7 signal per nucleus, which reflects the expression level of Pax7, revealed a significant decrease to 50% within 24 hours in the absence of Bmp7 (p = 0.005; n = 30; Figure 3M). We therefore conclude that satellite cells are competent to directly react to BMP signals by phosphorylation of Smad 1/5/8 under serum-free cultures. Furthermore, strong activation of BMP signaling seems to be sufficient to maintain Pax7 expression under these conditions.

### BMP signals inhibit differentiation of primary satellite cells

**Figure 4:**
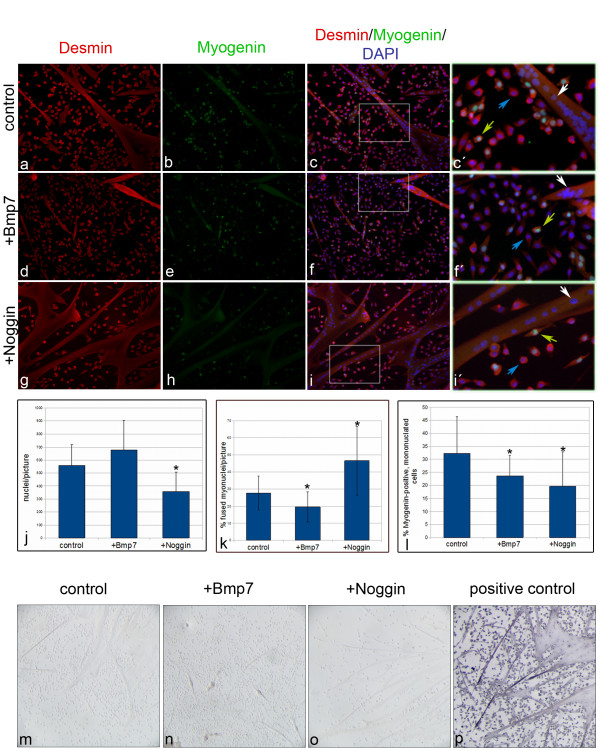
**BMPs regulate differentiation of primary myoblasts**. Myoblasts were treated with Bmp7 (D-F,N) or Noggin (G-I,O) for 3 days under differentiating conditions and double immunolabeled for Desmin (A,D,G) and Myogenin (B,E,H). Nuclei were counterstained with DAPI (C,F,I). Boxed areas in the overlay images (C,F,I) are enlarged in (C',F',I'). Myoblasts of different cell fates are present within cultures: fused (white arrow in C', F' and I'), mononucleated/Myogenin-negative (blue arrow in C',F',I') and mononucleated/Myogenin-positive (yellow arrow in C',F',I') cells. Myotube formation is reduced after Bmp7 stimulation (D-F) compared to control cells (A-C). Enhanced myotube formation was evident after Noggin treatment (G-I). Magnification is 100×. (J) Statistical analysis revealed a significant decrease in the total number of myonuclei upon Noggin treatment (n ≥ 13 individual cultures per treatment from 4 independent experiments). (K) Statistical analysis of the relative number of fused myonuclei (n > 13 individual cultures per treatment from 4 independent experiments) revealed a significant increase in Noggin-treated cultures compared to untreated controls (*p < 0.005; Wilcoxon test), while Bmp7 treatment lead to a significant decrease in the relative number of fused myonuclei (*p = 0.002; Wilcoxon test). (L) Analysis of the relative number of Myogenin-positive myonuclei within the population of mononucleated cells (n > 6 individual cultures per treatment from 2 independent experiments). (M-P) Primary satellite cell descendants do not express *alkaline phosphatase *in control (M), Bmp7 (N) and Noggin-treated cultures (O). Staining of a positive control is shown in (P). Magnification is 50×.

To further analyze the role of BMP signaling during satellite cell differentiation, we treated primary satellite cells cultured on matrigel under mitogen-restricted conditions with Bmp7 and Noggin. As Bmp7 treatment induced osteoblast differentiation in C2C12 cells we first analyzed the expression of the osteoblast marker alkaline phosphatase in parallel to Desmin, an intermediate filament protein specifically expressed in cells of the myogenic lineage (Figure 4; n ≥ 6). No expression of alkaline phosphatase could be detected (Figure 4M-P), demonstrating that, in contrasts to C2C12 cells, Bmp7 treatment does not induce osteoblast differentiation in primary satellite cells. Instead, all differentiating myoblasts from untreated (Figure 4A-C), Bmp7 (Figure 4D-F) and Noggin-treated cultures (Figure 4G-I) expressed the myoblast lineage marker Desmin (n ≥ 13). Furthermore, formation of MHC-positive myotubes could be detected under each culture condition (Additional file 1, Figure S1; n = 6).

To characterize the role of BMP signaling during the differentiation of primary satellite cells, we first counted the total number of nuclei from treated and control cultures. As expected, the total number of myonuclei in Noggin-treated cultures was significantly reduced (64%; p < 0.005) compared to untreated cultures, whereas it was increased after Bmp7 treatment (121%; p = 0.05; Figure 4J, n ≥ 13). Nevertheless, the relative number of fused myonuclei in Desmin-positive myotubes was decreased upon Bmp7 treatment (20%; p = 0.002), whereas Noggin treatment led to significantly enhanced myotube formation (47%) compared to untreated controls (Figure 4K; 28%; p < 0.005; n ≥ 13). Consequently, the fraction of mononucleated cells was significantly reduced upon Noggin treatment (data not shown).

As BMPs seem to keep C2C12 cells in a proliferating state we next investigated their effect on satellite cell proliferation by labeling S-phase nuclei at the end of a 3 day differentiation period for 2 hours with BrdU. MHC immunostaining was used to identify terminally differentiated myoblasts. The number of cells in S-phase was analyzed in undifferentiated, mononucleated cells, only. No statistically significant differences could be detected between the Bmp7-treated (15.1%, p = 0.11), the Noggin-treated (17.02%; p ≥ 0.1) and the control cultures (18.18%). It seems thus not likely that Bmp7 or Noggin treatment has major effects on cell cycle kinetics.

To estimate if treatment with Bmp7 or Noggin alters the number of cells committed to differentiate we next counted Desmin and Myogenin doublestained, mononucleated cells within the cultures. In Bmp7-treated cultures the relative number of Myogenin-positive, mononucleated cells was significantly reduced, strongly supporting the assumption that BMP signals inhibit the commitment to differentiation. Interestingly, after 3 days of Noggin treatment the relative number of Desmin- and Myogenin-positive, mononucleated cells was decreased as well (Figure 4L; n = 6). These data are in agreement with our hypothesis that BMP signaling keeps myoblasts proliferating and prevents the commitment to differentiation, whereas inhibition of BMP signaling supports commitment to terminal myoblast differentiation, leading to a rapid fusion of the myogenin-positive cells into myotubes.

**Figure 5:**
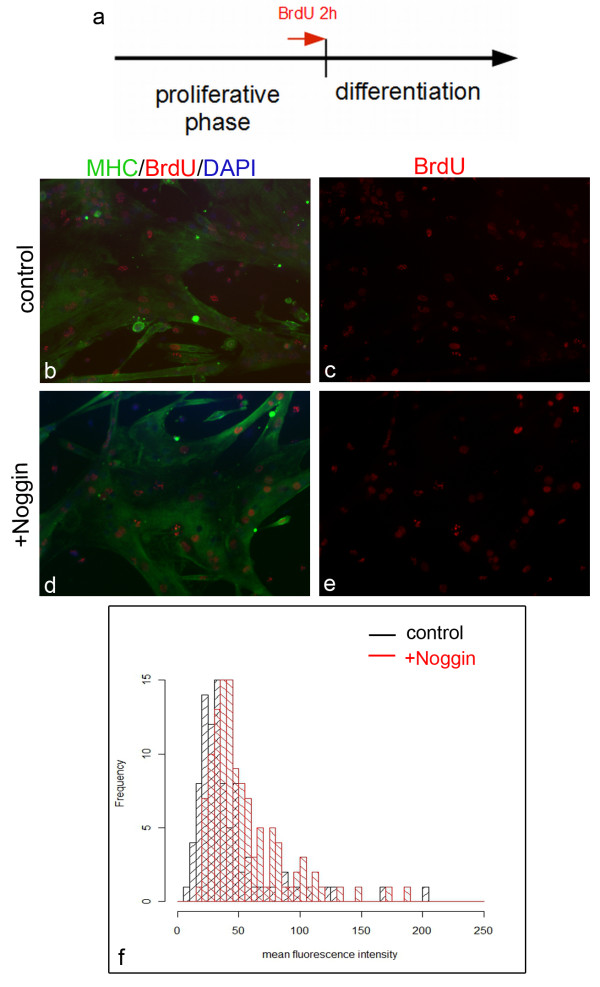
**Inhibition of BMP signaling triggers immediate cell cycle exit of satellite cells**. (A) Prior to the induction of differentiation, cultures of proliferating satellite cells were supplemented for 2 hours with BrdU and cultured for 3 days in differentiation medium. Myonuclei within MHC-positive myotubes from control cultures (B-C) show weak BrdU signals, while Noggin-treated myonuclei (D-E) show BrdU signals of stronger intensity in MHC-positive myotubes. (F) Analysis of the mean fluorescence intensity of the BrdU signal of Noggin-treated cultures (red columns; n = 92 myonuclei) compared to control cultures (black columns; n = 117 myonuclei). Histogram showing a shift in the frequency of Noggin-treated cells to higher levels of BrdU (p < 0,005; Wilcoxon test).

To further test if inhibition of BMP signaling triggers cell cycle exit we performed a BrdU pulse chase experiment in adherent satellite cell cultures (Figure 5). Directly before switching to differentiation medium, cultures of proliferating satellite cells were supplemented with BrdU for 2 hours. After removal of the BrdU-containing medium cells were differentiated for 3 days in control and Noggin containing medium (Figure 5A) and analyzed for BrdU labeling in fused, MHC-positive myotubes. As the BrdU pulse was received prior to the induction of differentiation, BrdU-positive myonuclei were found within the MHC-positive myofibers. Based on the idea that nuclei, which leave the cell cycle directly after the BrdU pulse, would retain a higher level of the BrdU signal than cells that keep proliferating and start to differentiate at later time points, we analyzed the intensity of the BrdU signal per nucleus (mean fluorescence/per nuclear area) within MHC-positive myotubes (Figure 5B-E). We then compared the number of highly and less intensively stained nuclei between the untreated and Noggin-treated cultures. In untreated cultures most myonuclei in MHC-positive myotubes exhibited a weak fluorescence signal indicating that myoblasts had undergone several cell divisions before fusing into myotubes (Figure 5B-C). In contrast, the proportion of nuclei displaying a more intense BrdU signal was significantly increased in Noggin-treated cultures (Figure 5D-F; p < 0.005; n ≥ 92). As BrdU labeling did not reveal any evidence that Bmp7 or Noggin treatment altered cell cycle kinetics in mononucleated cells (Additional file 1, Figure S1), these data further support our hypothesis that inhibition of BMP signaling stimulates cell cycle exit and differentiation of proliferating myoblasts.

### The p-Smad phoshorylation is lost in terminally differentiated myotubes

**Figure 6:**
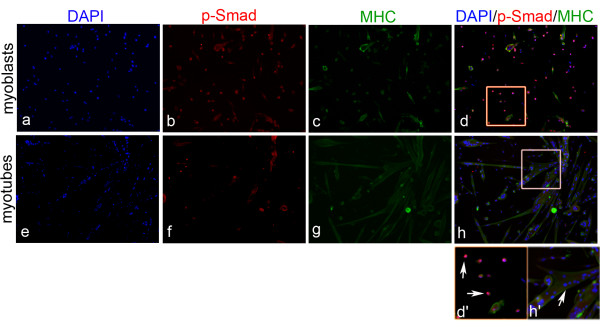
**The response to BMP signals decreases during late differentiation stages**. (A-H) Myoblasts were cultured for 9 days under differentiation conditions and double immunolabeled for p-Smad (B,F) and MHC (C,G). Nuclei were counterstained with DAPI (A,E). Boxed areas in the overlay images (D,H) are enlarged in (D',H'). p-Smad phosphorylation is strongly reduced in fused myotubes (white arrows in H') but is clearly present in mononucleated myoblasts (white arrows in d'). Magnification is 100×.

Our initial experiments indicated that nuclei in myofibers do not express p-Smad upon Bmp7 treatment. We thus asked if the BMP response is already turned off during the differentiation process. As the floating fiber culture model is not suitable to analyze later steps of the differentiation process we analyzed p-Smad in adherent satellite cell cultures during myotube formation and maturation (Figure 6). After culturing satellite cell descendants for 9 days under differentiating conditions most mononucleated and fusing myoblasts were present in the peripheral regions of clones (Figure 6A-D), whereas a high proportion of myoblasts had undergone fusion into MHC-positive myotubes within the center of the clones (Figure 6E-H). Whereas mononucleated and fusing cells were mostly p-Smad-positive most myonuclei within the myotubes were negative for p-Smad indicating that these ceased to respond to BMP signals or that BMP inhibitors were expressed during the differentiation process.

### Expression of a BMP inhibitor in primary satellite cell cultures *in vitro*

**Figure 7:**
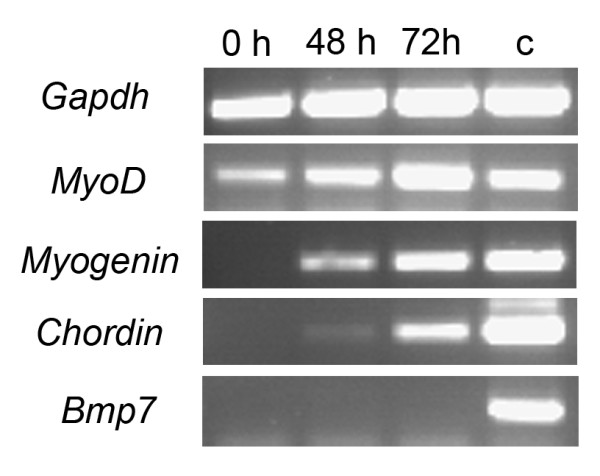
***Chordin *expression is upregulated in myofiber cultures**. *Gapdh*, *MyoD*, *Myogenin*, *Chordin *and *Bmp7 *expression was analyzed by RT-PCR of myofiber cultures at 0, 48 and 72 hours. cDNA of an E 12.5 embryo was used as a control. *Chordin *expression follows the expression of *Myogenin*.

As our data indicate a role for BMP signaling in fine-tuning proliferation and differentiation in satellite cells we compared the expression of BMP pathway components and BMP inhibitors to that of stage-specific myoblast markers in cultured satellite cells attached to myofibers for 0, 48 and 72 hours (Figure 7). On mRNA level, *MyoD *was already expressed in freshly isolated myofibers, although protein expression is not detectable at this stage [9,33]. *MyoD *expression increased with time reflecting the elevated number of satellite cells. *Myogenin *transcripts were first detected after 48 hours in culture and its expression was increased after 72 hours, a time point, at which the Myogenin protein could also be detected (Figure 2U). We next analyzed the expression of the most abundant BMP pathway components. We did not find expression of *Bmp2*, *Bmp4 *or *Bmp7 *under these conditions. Interestingly, while *Noggin *expression was below the detection limit, we found the BMP inhibitor *Chordin *weakly expressed at 48 hours and an increased expression at 72 hours (Figure 7 and data not shown). These data are consistent with the idea that Chordin, as an intrinsic inhibitor of BMP signaling, supports myoblast differentiation. Furthermore, increased *Chordin *expression might also contribute to the inhibition of BMP signaling and p-Smad phosphorylation observed in nuclei of fused myofibers (Figure 6).

### *In vivo *function of BMP signaling during muscle regeneration

**Figure 8:**
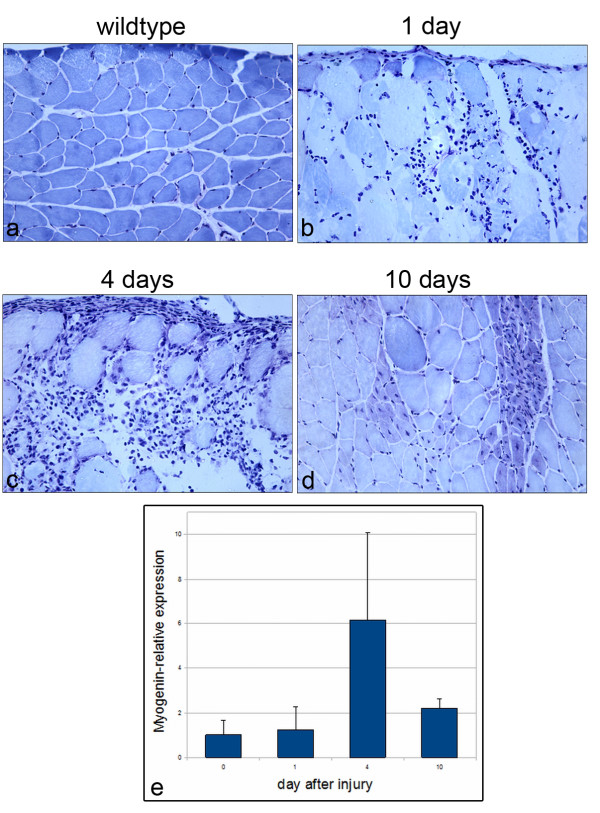
**Histological analysis of muscle after injury**. Sections of the gastrocnemius muscle before (A) and after trauma stained with Toluidin blue reveal invasion of mononucleated cells after 1 day (B) and massive regeneration after 4 days (C). 10 days after injury (D) regeneration is nearly completed and multiple regenerated fibers are visible. Magnification is 200×. (E) Expression analysis of *Myogenin *showing highest expression levels after 4 days of injury (n = 6 control animals, n = 2 animals at day 1 and n = 3 animals at day 4 and day 10, respectively).

**Figure 9:**
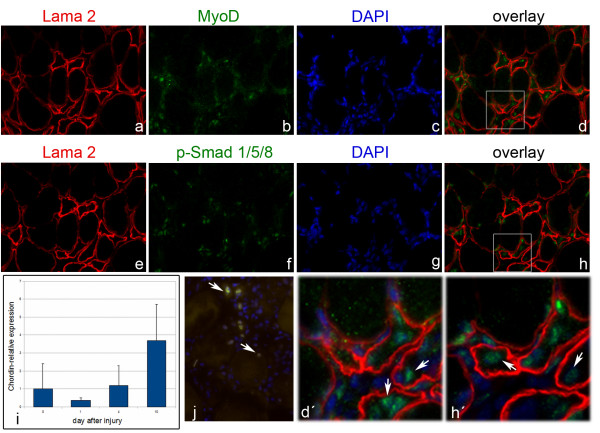
**Activated BMP signaling during muscle regeneration**. Parallel section ((A-D) and (E-H) represent images of 2 parallel sections) of the regenerating gastrocnemius muscle 4 days after injury were analyzed for Lama (A,E), MyoD (B) and p-Smad (F). Nuclei are stained with DAPI (C,G). Boxed areas in the overlay images (D,H) are enlarged in (D',H'). Both, MyoD (A-D,D') and p-Smad-positive nuclei (E-H,H') are located beneath the basement lamina of regenerating myofibers (white arrows in D',H'). (J) Double immunostaining of MyoD, detected with a goat anti-MyoD antibody (green), and p-Smad, detected with a rabbit anti-p-Smad antibody (red), revealed co-localization of both proteins in a subset of nuclei (yellow, arrows) in the regenerating tissue. Nuclei were counterstained with DAPI (blue). Magnification is 400×. (I) Analysis of *Chordin *expression by RT-PCR shows highest expression levels after 10 days of injury (n = 6 control animals, n = 2 animals at day 1 and n = 3 animals at day 4 and day 10).

In order to link our *ex vivo *observations to an *in vivo *function of BMP signals during muscle regeneration we analyzed regenerating gastrocnemius muscles of mice after induction of a blunt closed minor soft tissue trauma (Figure 8) [35]. Histological analysis of trauma tissue revealed the invasion of leukocytes into the injured region 24 hours after trauma induction (Figure 8B). After 4 days local myofiber degradation was visible, accompanied by the presence of numerous mononucleated cells within the same areas (Figure 8C). 10 days after injury myofiber morphology was restored and showed typical signs of regenerated muscle, like centrally located myonuclei and a small myofiber diameter (Figure 8D). Quantitative RT-PCR analysis of *Myogenin *expression during the regeneration process did not detect increased expression levels 24 hours after trauma compared to untreated controls (Figure 8E). A maximum of *Myogenin *expression was reached after 4 days, indicating that satellite cell descendants committed to differentiation were expanded in the injured muscle tissue. 10 days after trauma, the expression of *Myogenin *was decreased, but was still slightly elevated compared to controls. To analyze BMP signaling in activated satellite cells we used immunostaining against MyoD and p-Smad in parallel to Laminin α2 (Lama2), a protein of the basement lamina, which surrounds each myofiber including the attached satellite cells (Figure 9). As the highest level of regeneration seemed to occur 4 days after injury (Figure 8C), we chose this time point for our analysis. MyoD-positive cells underlying the basement lamina were present within regenerating regions (Figure 9A-D), demonstrating that 4 days after injury the majority of mononucleated cells were satellite cell descendants. Immunostaining for p-Smad together with Lama2 on parallel sections (Figure 9E-H) identified p-Smad-positive cells within the regenerating areas. Furthermore, similar to the MyoD-positive cells, these p-Smad-positive nuclei were located beneath the basement lamina of single myofibers and were thus likely to be satellite cell descendants. To further substantiate our data, we analyzed the expression of MyoD and p-Smad by double immunostaining using antibodies raised in different species (goat anti-MyoD and rabbit anti-p-Smad). We found both markers to be colocalized in a subset of nuclei representing activated satellite cells in the trauma region (Figure 9J). Together with the observation that postmitotic myonuclei of cultured myofibers were not stimulated by exogenous Bmp7 treatment, these data strongly support the hypothesis that activated satellite cells respond to BMP signals *in vivo *during muscle regeneration.

To investigate if BMP signaling is regulated endogenously during the regeneration process we analyzed the expression of BMPs and their inhibitors *in vivo*. We detected *Bmp2*, *Bmp4*, *Bmp7 *and *Noggin *expression in regenerating muscle tissue, but we did not find their expression increased upon trauma (data not shown). Interestingly, while at day 1 after trauma induction, when muscles were actively regenerating, *Chordin *expression was slightly reduced, its expression was upregulated at the end of the regeneration phase (day 10) (Figure 9I). It is thus likely that, similar as in the *ex vivo *cultures, BMP signaling is necessary for satellite cell expansion, while downregulation of BMP signaling triggers cell cycle exit and differentiation *in vivo.*

## Discussion

In this study we investigated the function of BMP signaling during satellite cell differentiation. Detailed analysis revealed that satellite cells respond to BMP signals during early stages of the activation process. BMP signals prevent cell cycle exit of activated satellite cells and proliferating myoblasts, leading to an expansion of the pool of satellite cell descendants. Furthermore, BMP signaling seems to support Pax7 expression at early activation stages. In contrast, inhibition of BMP signaling promotes cell cycle exit and differentiation leading to a reduced number of myoblasts and enhanced myotube formation. Interestingly, the BMP inhibitor *Chordin *is upregulated during the differentiation of primary satellite cell cultures. Inhibition of BMP signaling might thus support the transition from a proliferating to a differentiating state. To test our hypothesis *in vivo*, we analyzed muscle regeneration in a standardized injury mouse model. Similar to the *ex vivo *experiments we found BMP signaling upregulated in activated satellite cells at early stages of differentiation. At later stages, when *Myogenin *expression decreases in the trauma muscle we found *Chordin *expression upregulated. Based on these data we propose a role for endogenous BMP signals in expanding the pool of proliferating satellite cells and preventing premature differentiation into myotubes. A temporally coordinated, intrinsic inhibition of BMP signals by Chordin will subsequently regulate the pace of the differentiation process.

### Adult myogenesis recapitulate the embryonic program

Although BMP signals have been shown to regulate myogenesis during embryonic and fetal development, the role of BMP signaling during adult muscle regeneration is not well understood yet. Within the somite, Bmp4 from the lateral plate mesoderm and the overlying ectoderm has been reported to increase the number of Pax3-positive, proliferating myoblasts, whereas it reduces the number of *MyoD *expressing cells [36,37]. A detailed analysis revealed a feedback mechanism between Bmp4 and its antagonist Noggin, during which the expression of *Noggin *in the somites is induced by Bmp4 from the ectoderm. This feedback mechanism is thought to orchestrate proliferation and differentiation of muscle precursor cells restricting differentiation to cells in the deeper myotome [27]. Recently, BMPs have also been described to positively regulate the number of fetal myoblasts and myofibers during chick myogenesis [28]. Here we show that BMP signals play a similar role during satellite cell differentiation. Like during embryonic muscle development, activation of BMP signaling seems to keep primary myoblasts in an undifferentiated, mononucleated state, whereas inhibition of BMP signaling results in enhanced terminal differentiation of myoblasts into myofibers, thereby exhausting the pool of mononucleated cells. BrdU incorporation analyses in primary myoblast cultures did not reveal significant differences in the percentage of mononucleated cells in S-phase. It is thus not likely that BMP signaling plays a major role in regulating cell cycle kinetics. Instead our data are in agreement with the model that inhibition of BMP signaling triggers cell cycle exit and initiates the differentiation process. This model is further supported by the finding that the number of Myogenin-positive, mononucleated cells committed to differentiate is reduced after Bmp7 and Noggin treatment. We would thus propose that, like in the somite, BMP signals prevent the initiation of the differentiation program and the fusion into myofibers. In contrast inhibition of BMP signaling supports cell cycle exit, and commitment towards the differentiated myoblast, subsequently accelerating fusion into myofibers. Interestingly, it has recently been published that quiescent cells (reserve cells) can be detected in high density cultures of human fetal myoblasts [38]. Whether inhibition of differentiation and the subsequently increased cell density after BMP treatment will support such a reversion of cells into a quiescent state will be an interesting question for future studies.

### BMP signaling in satellite cells and osteoblast differentiation

Initially, multiple observations indicated that ectopic activation of BMP signaling in adult muscles is closely linked to ectopic bone formation. Most importantly, BMPs have been identified as 'Bone morphogenetic proteins', which induce ectopic bone formation if injected into rat muscle [22,39]. Furthermore, constitutive activating mutations of the type I BMP receptor, Acvr1, have been identified in the human syndrome Fibrodysplasia ossificans progressiva (FOP). FOP patients develop heterotopic ossifications in muscles and soft tissue upon injury [40,41]. Subsequent analysis of BMP signaling in the myogenic cell line C2C12 further demonstrated that C2C12 cells will differentiate into osteoblasts upon stimulation with BMP [22,24,25,39]. Recent gene expression studies revealed, however, that at least 25% of genes regulated in C2C12 cells are not differentially expressed in primary satellite cells [42]. Here we found that although BMP treatment inhibits myogenic differentiation in both, C2C12 and primary satellite cells, at least a subpool of C2C12 cells will subsequently express osteoblast markers, like Runx2 and alkaline phosphatase. In contrast, under the conditions chosen in our study, myoblast derived from primary satellite cells did not transdifferentiate into osteoblast-like cells. In agreement with these observations, two elegant studies have recently shown that not satellite cells, but a *Tie2 *expressing population of endothelial cells, contributes to the heterotopic ossifications observed in FOP lesions [43,44]. Nevertheless, although under *in vivo *conditions no bone formation is found in regenerating muscles, experiments using different cell culture conditions indicated that the differentiation potential of satellite cells includes the osteogenic cell fate *in vitro *[45].

### BMPs as inhibitors of satellite cells differentiation

During regeneration, satellite cells differentiate into myoblasts to restore the injured muscle and, in parallel, maintain the stem cell pool. It is therefore necessary that satellite cell proliferation, differentiation and fusion are tightly regulated. Here we show that BMP signaling plays a critical role in coordinating the differentiation of satellite cells from a proliferative to commited state. A proliferation promoting effect of BMPs has been reported for different progenitor cells [46,47]. This effect is, at least in part, mediated by members of the inhibitor of differentiation/inhibitor of DNA binding (ID) family of transcription factors, which are directly regulated by BMP signaling on a transcriptional level [48,49]. Id proteins act on the cell cycle machinery via different mechanisms to keep cells in a proliferating state and inhibit differentiation. Recently, it has been shown that deletion of *Id1/Id3 *impaired muscle regeneration in adult mice [50]. Furthermore, loss of these BMP-dependent transcription factors led to decreased proliferation in satellite cells. It is thus likely, that the inhibition of differentiation we observed upon BMP stimulation resulted from the activation of ID proteins.

In contrast to BMP treatment, depletion of BMP signals by Noggin induced fusion of myoblasts into myotubes. Interestingly, in our satellite cell cultures the relative number of Myogenin-positive, mononucleated myoblasts was reduced in Noggin-treated cultures compared to wild type cultures. Thus, inhibition of BMP signaling seems to not only induce commitment to the differentiation program, but to accelerate differentiation into fused myotubes in addition. Our pulse-chase BrdU labeling experiments further demonstrated that, upon inhibition of BMP signaling, highly labeled nuclei were found in the newly formed myotubes indicating that inhibition of BMP signaling induced cell cycle exit. Besides a direct effect on cell cycle exit, activation of the BMP pathway might result in a down-regulation of differentiation-promoting factors. Members of the MRF family of transcription factors, including MyoD and Myogenin, directly interact with Id proteins. This interaction prevents dimerization of MRFs with E-proteins and, thereby, the activation of differentiation-promoting target genes [51,52].

In this context it is interesting that we found the BMP inhibitor *Chordin *upregulated during the differentiation process *ex vivo *and *in vivo*. A Chordin-dependent intrinsic inhibition of BMP signals, which we mimicked by Noggin treatment in our experiments, might thus support cell cycle exit and differentiation if sufficient myoblast precursor cells have been generated. Whether *Chordin *expression is regulated by the environment, as for example by the number of progenitor cells, or by differentiation-dependent intrinsic transcription factors will be an important question to address in future studies.

In contrast to satellite cells and mononucleated myoblasts, fused myoblasts and nuclei of muscle fibers do not show overt phosphorylation of SMAD proteins, even upon Bmp7 treatment. This might reflect the endogenous production of BMP inhibitors by myotubes, which directly inhibit BMPs from binding to their receptors. At later stages, postmitotic cells might switch into a non-responive state by decreased expression of single BMP pathway components or continuous expression of inhibitors. Interestingly, when we cultured satellite cells under serum-free conditions without Bmp7 supplementation, p-Smad signals were not completely absent, although dramatically reduced compared to Bmp7-treated cells. The use of the artificial culture system does not exclude that BMPs endogenously produced by the satellite cells or fibers are sufficient to slightly activate the BMP signaling cascade in culture. In this context it is interesting to note that mechanical stress has been shown to trigger phosphorylation of BMP responsive SMAD proteins in other cell types like osteoblasts [53].

### Pax7 expression is maintained by BMP signaling

During chick embryonic myogenesis BMP signals derived from the ectoderm are required to maintain the pool of *Pax3 *expressing myoblast [26,27]. Here we show that in primary mouse satellite cells Pax7 expression is maintained by Bmp7 treatment. The sustained Pax7 expression might thus contribute to the Bmp7-induced delay in differentiation. In accordance with such a theory retroviral overexpression of Pax7 in primary satellite cell descendants results in a delay in satellite cell differentiation [54]. Pax7 has recently been shown to activate the expression of *Id3 *[55]. As Pax7 and p-Smad share at least one common target gene, both transcription factors might have a synergistic function to activate promoters of ID genes and other regulators of myoblast proliferation. Alternatively, as Pax7 has also been shown to be dispensable for adult muscle regeneration [56], the redundancy of these and other regulators of satellite cell proliferation might guarantee muscle regeneration under restricting conditions.

### BMP signaling is activated in regenerating muscle fibers *in vivo*

Recent studies have shown that *Bmp4 *and *Bmp6 *are expressed in quiescent satellite cells and are upregulated in C2C12 cells stably overexpressing Pax7 [55,57]. As we could not detect p-Smad proteins in quiescent satellite cells it is unlikely that p-Smad mediated BMP signaling contributes to the maintenance of the stem cell pool. The BMP inhibitor Chordin-like 2 (Chrdl-2), which has been described to be specifically expressed in quiescent satellite cells, might contribute to the inactivation of residual BMP signals [57].

Within our trauma model *Bmp2*, *Bmp4 *and *Bmp7 *expression was not increased. Nevertheless, p-Smad was clearly activated during the regeneration process. It is thus interesting to speculate that BMPs, like Hgf, are stored within the extracellular matrix and are released upon injury to trigger satellite cell expansion [14]. This would allow a fast, transcription-independent activation of the regeneration process. Similar to the *ex vivo *experiments we found an up-regulation of the BMP inhibitor *Chordin *in regenerating muscle tissue.

The *in vivo *data showed that *Myogenin *expression was upregulated before *Chordin* was expressed. Although Myogenin is often described as differentiation marker, it has been reported that Myogenin expression precedes cell cycle exit during myogenic differentiation [58]. Our data would thus indicate that upon commitment to myogenic differentiation the inhibition of BMP signaling by an intrinsic upregulation of *Chordin *will support cell cycle exit and terminal differentiation. In a parallel study *Noggin *has recently been described to be upregulated in trauma tissue [59] strongly supporting our hypothesis that fine-tuning of BMP signaling is a critical mechanism during the regeneration process.

### Interaction of BMP signals with other signaling factors

Our analysis has clearly shown that BMPs regulate skeletal muscle regeneration. Other trophic factors like Hgf, FGFs and TGFβ have also been shown to balance proliferation and differentiation of satellite cells, at least *in vitro*. The exposure of quiescent satellite cells, which express the Hgf-receptor c-met, to Hgf, lead to the activation of satellite cells and their re-entry into the cell cycle. Different lines of evidence indicate that *Hgf *is not expressed upon injury but is released from storage in the extacellular matrix allowing a fast induction of the regeneration process [13-15]. As p-Smad phosphorylation in satellite cells could only be detected after 24 hours of myofiber cultures, it is likely that BMP signals act downstream of Hgf-dependent activation of satellite cells. In addition, Hgf signals stimulate the expansion of the pool of satellite cell descendants and efficiently inhibit their differentiation [13,60,61]. In this function they are likely acting in parallel to BMP signals. Members of the FGF family of growth factors have also been implicated in promoting the proliferation of activated satellites cells. Like BMPs they seem to act downstream of Hgf [10,17,19]. It is interesting to note that at least three signaling systems interact to maintain the expansion of satellite cells. During the course of the differentiation process we observed the expression of the BMP inhibitor *Chordin*, which seems to support the initiation of the differentiation program. A similar function has been described for TGFβ signals, which inhibit proliferation and differentiation of satellite cells [19]. It will be an important task of future studies to decipher the epistatic relationship and the modes of interaction for these and other signaling systems in detail.

## Conclusions

**Figure 10:**
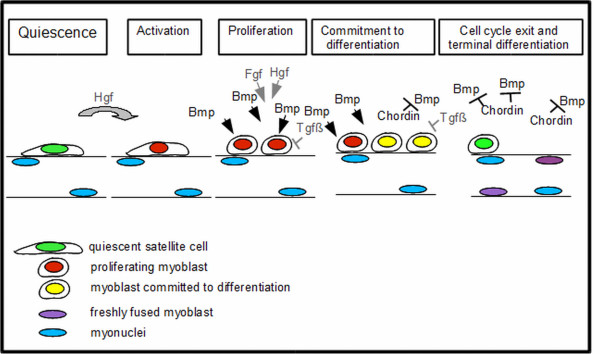
**Function of BMP signaling during satellite cell differentiation**. Quiescent satellite cells (green) do not respond to BMP signals. Upon activation, satellite cells express MyoD and respond to BMP signals with phosphorylation of p-Smad (red) and reduced differentiation. After satellite cell descendents are committed to differentiate (yellow) they express Myogenin and subsequently the BMP inhibitor Chordin. Upon inhibition of BMP signaling, myoblasts exit the cell cycle, fuse into myotubes and undergo terminal differentiation (purple). Postmitotic myonuclei (blue) irreversibly loose the competence to respond to BMP signals.

In summary, we propose a model (Figure 10), in which satellite cells are activated in a BMP-independent mechanism by Hgf. BMPs, possibly released from the extracellular matrix, will then act in parallel to Hgf and FGF signals and contribute to keep satellite cells in a proliferating state. This effect is likely to be driven by the activation of ID proteins by phosporylated SMAD proteins. After satellite cells are committed to differentiate they express increasing amounts of the BMP inhibitor Chordin. Inhibition of BMP signaling, likely in parallel with activated TGFβ signaling, will support cell cycle exit and initate the differentiation into myotubes. At later stages during the regeneration process fused myoblasts loose the competence to react to BMP signals possibly allowing the storage of BMP protein in the extracellular matrix.

## Materials and methods

### Mice

10-15 weeks old male C57BL6/J mice were bred and kept according to institutional guidelines. 8-10 weeks old male BALB/c mice were obtained from Harlan. Muscle trauma in anaesthetized BALB/c mice was induced as previously described [35]. Briefly, a drop-mass of 20 g (height 1.20 m) delivered a single impact on the posterior part of the gastrocnemius muscle without fracture of the bone. The trauma was induced on both hindlimbs. 3 animals were used per time point except 2 animals at day 1. Controls (n = 6) were anaesthetized only. Animals were sacrificed by cervical dislocation and muscle tissue was dissected for further processing.

### Single fiber isolation and culture

Single fiber isolation was carried out as previously described [62]. Mouse EDL muscles from C57BL/6 mice were carefully dissected and digested in 0.2% Collagenase type I (Sigma). Myofibers were released from the tissue by gentle tituration and transferred into DMEM/F12 medium containing 10% horse serum (Invitrogen) and 0.5% chicken embryo extract (MP Biomedicals) for up to 72 hours (floating myofiber cultures). Serum-free cultures were kept in DMEM/F12 medium containing 20% serum replacement 2 (Sigma). For satellite cell adhesion fibers were cultured in plating medium for 20 hours and each fiber was transferred into a single well of a 24-well plate coated with matrigel (BD Biosciences). After adhesion, proliferation medium containing DMEM/F12, 10% horse serum, 20% fetal bovine serum (Pan Biotech) and 1% chicken embryo extract was added to the culture and satellite cells were expanded for 4 days [63]. Differentiation was induced by switching to DMEM/F12 medium containing 10% horse serum, 2% fetal bovine serum and 0.5% chicken embryo extract. Cultures were supplemented with 100 ng/ml Bmp7 or 125 ng/ml Noggin (RnD systems). Medium and growth factors were replaced daily.

### C2C12 cell culture

C2C12 cells (DSMZ) were maintained subconfluent in DMEM containing 10% fetal bovine serum. For differentiation analysis 2 × 10^4 ^cells/cm^2 ^were seeded and grown for further 24 hours. Differentiation was induced by switching to DMEM containing 2% horse serum. Cultures were supplemented with 100 ng/ml Bmp7 or 125 ng/ml Noggin (RnD systems). Medium and growth factors were replaced every day. For proliferation analysis 10 μM 5-bromo-2'-deoxyuridine (BrdU) (Roche) was added to the culture 2 hour prior to fixation, unless otherwise mentioned (Figure 5).

### Tissue processing

Unfixed gastrocnemius muscles were carefully dissected from the hindlimb of injured mice and embedded in OCT (Sakura). Tissue samples were frozen in liquid nitrogen-cooled isopentane. 10 μm serial cryosections (Mikrom) were mounted on Superfrost slides (Roth) and dried for further processing. For histological staining OCT was removed and sections were stained in 0.1% Toluidin Blue (Merck).

### Immunostaining

For immunostaining, myofibers, satellite cell cultures and sections were washed in PBS and fixed in 4% paraformaldehyde (PFA). Cultures processed for BrdU detection were incubated in 2 N HCl for 30 minutes followed by neutralization in 0.1 M Na_2_B_4_O_7 _for 10 minutes. After washing, permeabilisation was carried out in 0.5% TritonX100/PBS (PBT) followed by blocking in 10% goatserum in 0.05% PBT. Antibodies diluted in blocking reagent were applied over night at 4°C. Secondary antibodies were applied for 45 minutes and followed by counterstaining with 100 ng/ml DAPI (Roth) in PBS for 5 minutes. Primary antibodies used were mouse anti-Pax7 and mouse anti-Myosin (clone A4.1025, DSHB), mouse anti-Myogenin (DAKO cytomation), rabbit anti-MyoD (Santa Cruz, except for Figure 9J), rabbit anti-phospho-Smad 1/5/8 and rabbit anti-cleaved Caspase-3 (Cell Signaling Technologies), rat anti-BrdU (Abcam), rabbit anti-Desmin (Sigma) in appropiate dilutions. Detection of primary antibodies was carried out using Alexa 488 and Alexa 568 fluorochrome-coupled secondary antibodies (Molecular Probes). For tissue double labeling (Figure 9J) goat anti-MyoD (Santa Cruz, N-19) and rabbit anti-phospho-Smad 1/5/8 (Cell Signaling Technologies) were used. Secondary detection was carried out using Alexa-fluorochrome-coupled secondary antibodies (Molecular probes) raised in donkey.

### Alkaline phosphatase activity assay

Adherent cells were washed in PBS and fixed in 4% PFA for 10 minutes. After several washes in PBS, cells were preincubated in 100 mM Tris pH 9.5, 100 mM NaCl and 50 mM MgCl_2 _(NTM). NBT/BCIP substrate (Roche) was diluted in NTM and applied to the cells for 45 minutes. Internal positive control on primary cells was carried out by incubating fixed cells with a Fgf8-alkaline phosphatase fusion protein. Cells were treated as described above.

### RNA isolation and PCR analysis

RNA was isolated using Trizol (Invitrogen) and cDNA was prepared using Superscript III (Invitrogen) according to the manufacturer's instructions. Quantitative PCR was performed on a Step one plus realtime PCR system (Applied Biosystems) using EVA green PCR master mix (Biobudget) according to the manufacturer's guidelines. β2-miroglobulin was used as reference. Relative quantification was carried out using the method of Pfaffl [64]. Semi-quantitative PCR was carried out using DreamTaq Polymerase (Fermentas) on Biometra cycler. Primers used were as follows: *Gapdh *for: 5'CCAGGAGCGAGACCCCACTA3'; *Gapdh *rev: 5'GCAGTTGGTGGTGCAGGATG3'; *β2 m *for: 5'GCTCGGTGACCCTGGTCTTT3'; *β2 m *rev: 5'GAGGCGGGTGGAACTGTGTT3'; *MyoD *for: 5'CGAGCACTACAGTGGCGACTCAGAT3'; *MyoD *rev: 5'GCTCCACTATGCTGGACAGGCAGT3'; *Myogenin *for: 5'CCATCCAGTACATTGAGCGCCTACA3'; *Myogenin *rev: 5'ACGATGGACGTAAGGGAGTGCAGAT3'; *Chordin *for: 5' ATAGCCCTGCTCACCCTC AGTGACA3'; *Chordin *rev: 5'GCTCCAACCAGTCCATCTCTTGGTC3'; *Bmp7 *for: 5'CCTGTCCATCTTAGGGTTGC; *Bmp7 *rev: 5'CAGTGAGGAAGTGGCTGTCC3'.

### Imaging and statistical analysis

Fluorescence pictures were taken on a Zeiss Axiovert 200 microscope with a Spot 23.0 camera (Diagnostic Instruments) and Metamorph Imaging Software (Visitron Imaging systems). For each experiment done in triplicates, 10 pictures/well were randomly taken at 200-fold magnification. The Metamorph cell cycle tool was used to count nuclei and BrdU or Myosin-positive C2C12 cells. For primary satellite cell descendants, 2 individual pictures from the middle of each clone were taken in a 100-fold magnification and cells were counted and categorized manually. Quantification of fluorescence signal intensity was performed using the Metamorph image analysis tool. In short, the fluorescence intensity in the nucleus was measured and related to the total area of the nucleus. Statistic significance was assessed using the generalized Wilcoxon test (for Myosin-positive cells) or Wilcoxon test (for all other experiments). p-values < 0.05 were regarded as significant. Diagrams show mean values. Bars indicate standard deviation.

## Authors' contributions

MF carried out the experiments, statistical evaluation and drafted the manuskript. AV and MW participated in designing the study, evaluation of data and drafting the manuscript. SBF and FW induced the muscle trauma. SS contributed to the analysis of the trauma muscle. All authors have read and approved the manuscript.

## Supplementary Material

Additional file 1 **Figure S1 BMP signaling does not alter cell cycle kinetics of myoblasts**. Myoblasts were differentiated in control medium (A-C,C) or in medium supplemented with Bmp7 (D-F,F) or Noggin (G-I,I) for 3 days and labeled for two hours with BrdU at the end of the cultivation phase. (J) No statistically significant alteration of the percentage of cells in S-phase within the pool of mononucleated myoblasts could be detected after Bmp7 (p = 0.11) or Noggin (p = 0.66) treatment (n > 6 individual cultures per treatment from 2 independent experiments). Boxed areas in (C,F,I) are enlarged in (C',F',I'). Magnification is 100×. Click here for file
