# Supplementary figures and images for: BMP signaling balances proliferation and differentiation of muscle satellite cell descendants
Source: BMC Cell Biol. 2011 Jun 6;12:26. doi: 10.1186/1471-2121-12-26 (PMC3149017; doi:10.1186/1471-2121-12-26)

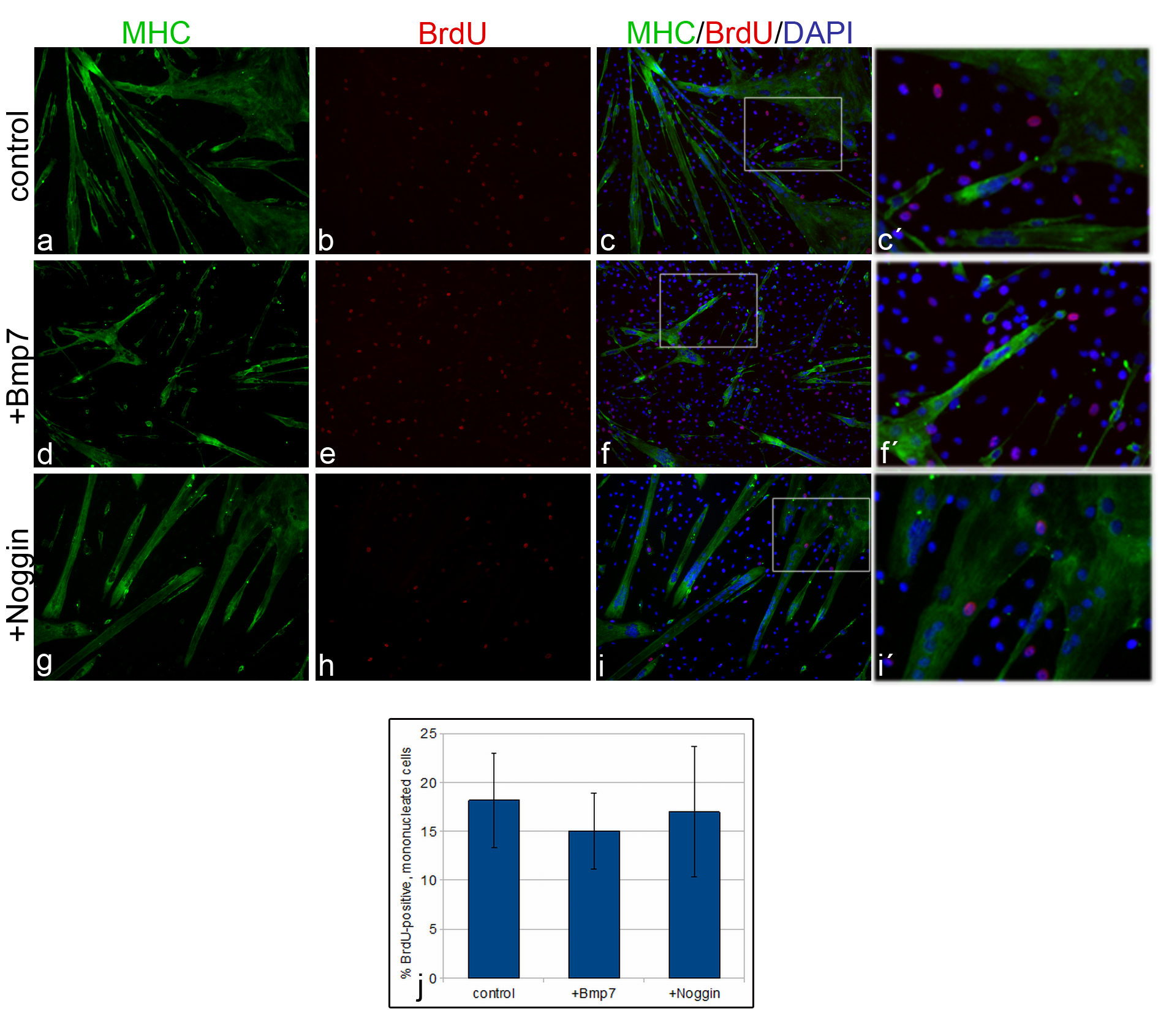

Supplement: Additional file 1 — Figure S1 BMP signaling does not alter cell cycle kinetics of myoblasts. Myoblasts were differentiated in control medium (A-C,C) or in medium supplemented with Bmp7 (D-F,F) or Noggin (G-I,I) for 3 days and labeled for two hours with BrdU at the end of the cultivation phase. (J) No statistically significant alteration of the percentage of cells in S-phase within the pool of mononucleated myoblasts could be detected after Bmp7 (p = 0.11) or Noggin (p = 0.66) treatment (n > 6 individual cultures per treatment from 2 independent experiments). Boxed areas in (C,F,I) are enlarged in (C',F',I'). Magnification is 100×. [file 1471-2121-12-26-S1.TIFF]
